# Association between uncontrolled diabetes and periodontal disease in US adults: NHANES 2009–2014

**DOI:** 10.1038/s41598-023-43827-y

**Published:** 2023-10-04

**Authors:** Giang T. Vu, Shaminul Shakib, Christian King, Varadraj Gurupur, Bert B. Little

**Affiliations:** 1 School of Global Health Management and Informatics, University of Central Florida, Orlando, FL 32801 USA; 2 Department of Health Management and Systems Sciences, University of Louisville, Louisville, KY USA

**Keywords:** Dentistry, Dental public health, Dental epidemiology

## Abstract

This study examined the relationship between uncontrolled diabetes and periodontal disease (PD) among adults in the United States. We used data from the 2009–2014 National Health and Nutrition Examination Survey (NHANES) with a sample of 6108 adults ages 30 and over. To measure PD status, we used the Centers for Disease Control and Prevention/American Academy of Periodontology’s standards. To classify DM status (no DM, DM with HbA_1c_ < 9%, diabetes with HbA_1c_ ≥ 9%),we used self-reported Diabetes Mellitus (DM) diagnosis and laboratory report of HbA_1c_. Approximately 8.5% of the sample had controlled DM, and 1.7% had uncontrolled DM, for a total of 10.2% DM in the analysis. Multivariate logistic regression showed that compared to those without DM, PD was significantly increased with controlled DM (adjusted odds ratio (aOR) = 1.32, 95% confidence interval (CI) 1.01–1.73, p < 0.05) and even more with uncontrolled DM (aOR = 2.48, 95% CI 1.52–4.04, p < 0.001), after adjusting for covariates. Factors that reduced the prevalence of PD included annual dental visits, female gender, and college education. Factors that significantly increased PD prevalence were cigarette smoking, non-white race, income < 200% Federal Poverty Level, and older age (age > 50 years). In conclusion, uncontrolled DM was significantly associated with higher odds of PD among adults in the US.

## Introduction

Diabetes mellitus (DM) is the seventh leading cause of death in the United States (US)^1^. The prevalence of DM has increased dramatically over the past three decades, increasing from less than 5% in 1990 to 12.4% in 2012^1^. It is predicted that one in three people in the US will have DM by 2025^2^. Globally, the prevalence of DM is expected to reach 10.9% (700 million) by 2045^3^. Individuals with DM have particularly elevated blood glucose, which may lead to a higher prevalence of diseases associated with chronic inflammation, including periodontitis^4^.

Periodontal disease (PD) is highly prevalent in the US with an estimated 42% of adults in the US having it^5^. PD is characterized by periodontal pocket formation, loss of gingival attachment, and alveolar bone resorption beneath the soft tissue^6^. Chronic inflammation, such as PD, is associated with increased insulin resistance, adversely affecting glycemic control^4^. DM and PD are two highly prevalent chronic diseases, and empirical evidence suggests that there is a link between the two^7^. Those with DM have a higher likelihood of developing PD. A large body of literature has demonstrated that periodontitis is an oral complication of DM, which severely exacerbates the development, progression, and severity of PD^8^. One biological plausibility is that diabetic patients, especially those with high levels of HbA_1c_, are more susceptible to infections and impaired wound healing^9^. Hyperglycemia in uncontrolled diabetes may lead to increased levels of glycation end products that will cause negative impacts on oral soft-tissue inflammation, endothelial cell changes, impaired bone metabolism, and subsequently periodontal health^10^.

Previous studies demonstrated a bidirectional relationship between DM and PD and reported that PD is more severe among people with DM^11^. Increased severity of PD negatively influences glycemic control in DM by increasing insulin resistance^12^. PD treatment may improve periodontal status, but it is unknown whether this improves glycemic control among DM patients^2^. A study conducted by Garcia et al. using data from the 2009–2014 National Health and Nutrition Examination Survey (NHANES) found that PD was not associated with self-reported DM and on the contrary was associated with HbA1c levels^2^. Another study using the same data but different encoding of DM status reported an association between PD with uncontrolled DM among US adults^12^. However, this study did not account for potential confounders related to dental preventive care (e.g., annual dental visit, reason for a dental visit), which are factors known to be significant predictors of PD^12^. The prevalence of periodontitis was greater among those with DM (59.9%) compared to non-DM individuals (40.4%)^5^.

More recent population-based surveys with more comprehensive data collection and modeling are needed to more precisely define the relationship between PD and glycemic control among US adults with DM. Therefore, the objectives of the present study are to analyze the association between PD and uncontrolled DM among adults in the US using periodontitis surveillance data from NHANES 2009–2014, and analyze factors related to uptake of dental preventive care among DM individuals.

## Methods

### Data source

We used publicly available data from the 2009–2014 NHANES collected by the Centers for Disease Control and Prevention (CDC)^13^. NHANES uses a cross-sectional, multistage probability cluster sample design to obtain a representative sample of the US civilian, noninstitutionalized population^5,14^. Sampling probability weights were used to balance representativeness of the sample, allowing extrapolation to the US population, and correct possible biases introduced in population sampling.

### Study population

The study sample included 6108 adults ≥ 30 years old who received a full-mouth periodontal examination^15^. Participants in the study were sampled to be representative of 143.8 million U.S. adults in a probability-weighted sample^5^. Trained and calibrated dentists conducted dental examinations in mobile examination centers (MEC)^5^. Participants with missing information on one or more variables of interest were excluded from the analysis using listwise deletion.

### Definition and outcome

Presence of PD (0—no, 1—yes) was the primary outcome variable. Participants were categorized with PD if there was clinical attachment loss (CAL) and their periodontal pocket depth met the standards established by the CDC/AAP standard for population-based periodontitis surveillance^5^. PD is defined as having two or more interproximal sites with clinical attachment loss (CAL) of 3 mm (on different teeth) in addition to two or more interproximal sites with periodontal probing depth (PPD) of 4 mm (not on the same tooth), or at least one interproximal site with PPD of 5 mm.

### Independent variables

In this study, the DM status was used as a key predictor which included the following categories: (i) no DM, (ii) DM with controlled glycated hemoglobin [HbA_1c_], and (iii) DM with uncontrolled HbA_1c_ (≥ 9). DM status was determined based on the individual’s self-reported DM questionnaire and laboratory determined HbA_1c_. Self-reported DM status was collected using the following question: "Other than during pregnancy, have you ever been told by a doctor or health professional that you have DM?" Participants who responded with a “no” were considered non-DM, and those who responded with a "yes" and had an HbA_1c_ of less than 9% were classified as “controlled DM”^2,16,17^. Participants who responded "yes" and had an HbA_1c_ ≥ 9% were classified as “uncontrolled DM”^2,16,17^. DM status was defined by simultaneously using a binary DM (yes, no) and HbA_1c_ level (< 9%, ≥ 9%) variables to determine the degree of control^2,17^.

The following confounding factors included based upon prior publications include: (i) dental visit (ii) dental floss (iii) smoking status (iv) education (v) family income (vi) gender (vii) race/ethnicity, and (viii) age were selected based on previous epidemiologic studies^18–21^. Dental visits were categorized into two categories: ≤ 12 months and > 12 months. Dental visits were based on reported prophylaxis or annual/biannual examinations, dental pain or a dental procedure. Participants who flossed at least once a week, were classified as using dental floss. Age was recoded into three categories: young (30–44 years old), middle aged (45–64 years old), and elderly (≥ 65 years old). Gender was categorized as male or female. Participant race and ethnicity were coded as non-Hispanic White, non-Hispanic Black, Hispanic, and other race. Two educational level categories (high school diploma or lower, college or above) were coded^4,12,14^. The federal poverty level (FPL) was divided into two categories: those with incomes below 200% FPL and those with incomes at or over 200% FPL^2,14^. Smoking status was also considered and classified as never smokers, former smokers, or current smokers^5^. Participants who had smoked every day or some days with at least 100 cigarettes in the past were categorized as current smokers^5^. Participants who reported currently not smoking but having smoked at least 100 cigarettes were considered as former smokers^5^. Participants who smoked fewer than 100 cigarettes in the past were defined as never smokers^5^.

### Statistical analysis

Data analysis was carried out using SAS (Version 9.4, SAS Institute, Cary, NC, USA) and R software (version 3.6.3) with the survey package (R Foundations). Weighted point estimates were used to make our findings representative of the US population in this manuscript. It is important to inform the reader that adult population in the US was used in the sampling weighted statistical calculations of MEC examination with cluster and stratum variables provided by the NHANES^5,14^.

Chi-squared tests were used to analyze the prevalence of periodontal disease by explanatory variables. The relationship between PD and DM were further analyzed using multiple logistic regression. The adjusted odds ratios (aOR) with 95% CIs were reported, and the significant level of p values was less than 0.05.

## Results

### Sample characteristics

The study sample included 6,108 individuals aged 30 years and older (Table 1). The unadjusted prevalence of PD was 38.7% (95% CI 35.3–42.1). The mean age of the study participants was 51.7 years old (SD = 14.2 years). Uncontrolled DM was present among 1.7% of participants while 8.5% and 89.8% of participants had controlled or no self-reported DM diagnosis, respectively. An estimated 66.2% of the participants had annual dental visits. Routine daily dental flossing was reported by 73.9% of participants. Approximately 17.8% of participants reported to currently smoke cigarettes. Nearly one-third of the study sample (31.8%) earned less than 200% FPL, and an estimated 33.5% of participants have at most a high school degree. About half of the participants (51.2%) were female, and 69.4% were white. Those older than 45 represented 62.6% of study participants.

**Table 1:** Prevalence of periodontal disease among U.S. adults.

	n	Weighted estimate, percentage (95% CI)	Periodontal disease presence, percentage (95% CI)	Periodontal disease absence, percentage (95% CI)
Overall	6108	100%	38.7 (35.3–42.1)	61.3 (57.8–64.7)
Age in years (SD)	51.65 ± 14.24	50.86 ± 13.55		
Diabetes status				
No diabetes	5321	89.8 (88.8–90.8)	36.6 (33.3–40.0)	63.3 (60.1–66.7)
Controlled diabetes	648	8.5 (7.6–9.4)	55.0 (50.0–60.5)	45.0 (39.5–50.5)
Uncontrolled diabetes	139	1.7 (1.3–2.1)	67.7 (58.2–77.3)	32.3 (22.8–41.8)
Dental visit				
> 12 months	2412	33.8 (31.2–36.5)	52.9 (48.9–56.9)	47.1 (43.1–51.1)
≤ 12 months	3696	66.2 (63.6–68.9)	31.5 (28.2–34.7)	68.6 (65.3–71.8)
Dental floss				
No dental flossing	1839	26.1 (24.5–27.8)	48.7 (44.4–53.1)	51.2 (46.9–55.6)
Dental flossing	4269	73.9 (72.3–75.5)	35.2 (31.9–38.5)	64.8 (61.5–68.1)
Smoking				
Never smoker	3449	56.0 (54.0–57.8)	30.1 (26.5–33.6)	70.0 (66.4–73.5)
Former smoker	1524	26.3 (24.4–28.1)	42.7 (38.5–47.0)	57.3 (53.0–61.5)
Current smoker	1135	17.8 (16.4–19.1)	60.1 (55.7–64.4)	39.9 (35.6–44.3)
Education				
High school or less	2503	33.5 (30.1–37.0)	56.1 (53.0–59.3)	43.9 (40.7–47.1)
College or above	3605	66.5 (63.0–69.9)	30.0 (26.9–33.1)	70.1 (67.0–73.2)
Income				
Income ≥ 200% FPL	3409	68.2 (64.6–71.9)	31.7 (28.3–35.1)	68.3 (64.9–71.7)
Income < 200% FPL	2699	31.8 (28.1–35.4)	53.8 (49.8–57.9)	53.8 (49.8–57.9)
Sex				
Male	2968	48.9 (47.4–50.2)	45.7 (42.0–49.4)	54.3 (50.6–58.0)
Female	3140	51.2 (49.8–52.6)	32.1 (28.4–35.8)	68.0 (64.3–71.6)
Race				
White	2569	69.4 (64.9–73.9)	33.6 (29.8–37.4)	66.4 (62.6–70.2)
Hispanic	1261	12.8 (9.6–16.1)	51.8 (48.0–55.7)	48.1 (44.3–52.0)
Black	1337	10.3 (8.0–12.5)	53.5 (48.5–58.6)	46.5 (41.4–51.5)
Others	941	7.5 (6.3–8.7)	43.4 (37.2–50.0)	56.6 (50.4–62.8)
Age				
Young (30–44 years)	2271	37.3 (34.9–39.9)	27.5 (23.6–31.3)	72.5 (68.7–76.4)
Middle-aged (45–64 years)	2577	45.2 (43.0–47.5)	41.8 (37.8–45.7)	58.2 (54.3–62.2)
Elder (≥ 65 years)	1260	17.4 (15.6–19.1)	55.0 (49.5–60.5)	45.0 (39.5–50.5)

Diabetes status included three categories: (1) no diabetes, (2) DM with HbA1c level < 9% that we categorized as controlled diabetes, and (3) DM with HbA1c level that we categorized as uncontrolled diabetes.

Dental visits had two categories: ≤ 12 months and > 12 months;

Dental floss: If participants flossed at least once a week, they were classified as using dental floss.

Smoking status was also considered and classified as never smokers, former smokers, or current smokers. Participants who had smoked every day or some days with at least 100 cigarettes in the past were categorized as current smokers. Participants who reported currently not smoking but having smoked at least 100 cigarettes were considered as former smokers. Participants who smoked fewer than 100 cigarettes in the past were defined as never smokers.

*US* United States, *CI* confidence interval, *n* number of participants, *SD* standard deviation, *FPG* federal poverty levels, *DM* diabetes mellitus.

Bivariate analysis (Table 1) showed that adults with controlled and uncontrolled DM had a higher prevalence of PD (55.0% and 67.7%, respectively) than those without DM (36.6%). Participants who had an annual dental visit had a lower prevalence of PD (31.5%) than those who did not have an annual dental visit (52.9%). Similarly, participants who flossed daily had a PD lower rate (35.2%) than those who did not (48.7%). The prevalence of PD was highest among current smokers (60.1%), followed by past smokers (42.7%), and non-smokers (30.1%). The prevalence of PD was lower among college educated participants (30%) compared to participants who had at most a high school degree (56.1%). PD was more prevalent (53.8%) among participants with incomes below the FPL of 200% compared to participants with incomes above the FPL of 200% (31.7%). Females had a lower PD prevalence (32.1%) than males (45.7%). Whites had the lowest PD prevalence (33.6%), followed by other races (43.4%), excluding Hispanics (51.8%) and Blacks (53.5%). Middle-aged and older adults had a higher PD prevalence (41.8 and 55.0%, respectively) than younger adults (27.5%).

### Uncontrolled diabetes and periodontal disease

Multiple logistic regression analysis (Table 2) indicated that participants with uncontrolled DM had 3.56 higher odds (95% CI 2.61–4.94) of having PD compared to those with no PD, adjusted for annual dental visits, dental flossing, smoking status, education level, income, sex, race, and age. Annual dental visits significantly lowered the odds of developing PD by 2.38 (0.42; 95% CI 0.39–0.47). Current and former smokers had a higher likelihood of developing PD (2.65 and 1.60, respectively) compared to non-smokers. Participants with a college degree or more had 2.70 lower odds (0.37; 95% CI 0.34–0.40) of developing PD compared to those who had at most a high school degree. The likelihood of developing PD increased by 2.19 times (95% CI 2.02–2.38) if income was below 200% of FPL. Females had (0.48; 95% CI 0.45–0.52) lower odds of developing PD than males. Minorities had higher odds of PD than whites (aOR_Hipanic_ = 1.90, aOR_Black_ = 2.07, aOR_others_ = 1.20), and older adults were more likely than younger adults to have PD (aOR_middle-aged_ = 2.34, aOR_elder_ = 3.69).

**Table 2:** Multiple logistic regression analysis of periodontal disease among US adults.

	Adjusted odds ratio (aOR)	*p* value
Diabetes status		
No diabetes	Ref	
Controlled diabetes	1.96 (1.72–2.24)	0.047
Uncontrolled diabetes	3.56 (2.61–4.94)	< 0.001
Dental visit		
> 12 months	Ref	
≤ 12 months	0.42 (0.39–0.47)	< 0.001
Dental floss		
No dental flossing	Ref	
Dental flossing	0.50 (0.46–0.55)	0.159
Smoking		
Never smoker	Ref	
Former smoker	1.60 (1.46–1.76)	< 0.001
Current smoker	2.65 (2.38–2.94)	< 0.001
Education		
High school or less	Ref	
College or above	0.37 (0.34–0.40)	< 0.001
Income		
Income ≥ 200% FPL	Ref	
Income < 200% FPL	2.19 (2.02–2.38)	< 0.001
Sex		
Male	Ref	
Female	0.48 (0.45–0.52)	< 0.001
Race		
White	Ref	
Hispanic	1.90 (1.72–2.09)	< 0.001
Black	2.07 (1.86–2.29)	< 0.001
Others	1.20 (1.06–1.36)	< 0.001
Age		
Young (30–44 years)	Ref	
Middle-aged (45–64 years)	2.34 (2.14–2.55)	< 0.001
Elder (≥ 65 years)	3.69 (3.31–4.12)	< 0.001

Diabetes status included three categories: (1) no diabetes, (2) DM with HbA1c level < 9% that we categorized as controlled diabetes, and (3) DM with HbA1c level that we categorized as uncontrolled diabetes.

Dental visits had two categories: ≤ 12 months and > 12 months.

Dental floss: If participants flossed at least once a week, they were classified as using dental floss.

Smoking status was also considered and classified as never smokers, former smokers, or current smokers. Participants who had smoked every day or some days with at least 100 cigarettes in the past were categorized as current smokers. Participants who reported currently not smoking but having smoked at least 100 cigarettes were considered as former smokers. Participants who smoked fewer than 100 cigarettes in the past were defined as never smokers.

*US* United States, *aOR* adjusted odds ratio, *CI* confidence interval, *n* number of participants, *SD* standard deviation, *FPG* federal poverty levels, *Ref* reference category, *DM* diabetes mellitus.

## Discussion

The bidirectional relationship between PD and DM has been studied extensively over several decades^11^. The current analysis aligns with the literature that uncontrolled DM was associated with higher likelihood of PD among US adults, which was measured objectively by trained dental and medical professionals in the NHANES examination^2,5,12^. Results of the present study indicate that participants with uncontrolled DM were more likely to have PD than those with controlled DM or non-DM. Individuals with controlled DM had higher odds of PD than non-DM individuals.

This finding is consistent with analyses showing that the odds of having PD was greater among DM individuals and elevated HbA_1c_ compared to non-DM individuals^2^. Most studies used either HbA_1c_, fasting plasma 2-h glucose challenge, or self-reported DM, and found a significantly increased odds ratio of PD with hyperglycemia^2,5,12^. Interestingly, when HbA_1c_ (or fasting plasma glucose) and self-reported DM were used separately as two variables in analyses, only one of them had a significant relationship with PD. For example, an analysis of the 2009–2014 NHANES data reported that self-reported DM status was not significantly associated with periodontitis despite the large sample size^2^. A possible explanation is that some individuals with DM control their glucose sufficiently close to lower their likelihood of having PD, which is not reflected in the binary variable. HbA_1c_ captures individual levels of glycemic control, and better reflects the relationship between variation in glycemia and PD. For example, there is evidence of a significant linear relationship between laboratory determined HbA_1c_ and clinically measured PD^2^. Specifically, they demonstrated that the prevalence of PD increases with elevated HbA_1c_ levels. Objective measurement of the severity of DM improves the accuracy of the analysis of glycemic management that cannot be achieved with a binary response variable.

Using both DM indicator variables (self-reported DM and laboratory HbA_1c_) separately would be redundant measurements and produce multicollinearity in the model. For instance, if a participant had HbA_1c_ levels under 9%, they could be considered as either having uncontrolled DM or no DM. If a participant had HbA_1c_ levels greater than 9%, they could be either in the early stage of their diabetics or gestational diabetes. We need to use an additional measurement such as DM self-reported diagnosis (yes = 1, no = 0) to confirm DM status and help reduce selection bias (by excluding gestational diabetes). Using only HbA_1c_ levels (such as < 6.5) would not confirm the absence of DM if it were being successfully treated. The diagnosis of having DM needs to be confirmed by a physician. Unlike previous classification schemes, a three-category variable was used in the present study to avoid redundancy in measurement of DM. To the best of our knowledge, the present study is one of the few studies^2,17,22^ using a hybrid variable of three-categories to measure DM status in dental research. We found significant relationships between PD with controlled and uncontrolled DM.

PD is a well-known complication of DM^4^. Hyperglycemia plays an important role in PD^23^. Previous studies found that the level of glycemic control in DM has a large impact on the severity of PD^16^. Hyperglycemia induced inflammasome activation causes gingival epithelium^7^. Simultaneously, hyperglycemia accelerates the damage to the gingival epithelial barrier, impairing its function^7^. Hypoglycemic drugs (e.g., metformin, glipizide, gliflozin) may help decrease the prevalence of PD in individuals with DM by lowering glycemia, and the attenuation of oxidative stress and inflammation^23^. Given the bidirectional relationship between PD and DM, it is challenging to determine the sequence of occurrence between these two diseases due to their mutual interactions^7,10^. Instead of focusing on solely one disease, it is necessary to focus on disease management and treatment guidelines for both conditions. In addition to blood glucose management, it is important to emphasize the use of preventive dental services and plaque control^24,25^. Preventive dental care utilization is associated with improved health care outcomes and reduced average costs for low-income patients with diabetes^24^. Disease management for both conditions is even more critical for individuals with severe diabetic comorbidities, especially older adults who are unable to independently manage their oral health^9,12^.

Consistent with previous studies^2,5,12^, the social determinants of health (SDOH) such as older age, male gender, lower education level, lower income, racial/ethnic minorities, and smoking were significantly associated with higher odds of PD, after adjusting for covariates. Specifically, elder and middle-aged adults have significantly increased odds of having PD, 3.69 and 2.34 fold, respectively, compared to young adults. This finding emphasizes the need for increased dental coverage in the general public^25^. Another important finding is that adults with annual dental visits had a significantly lower likelihood of periodontitis, which supports the Healthy People 2023’s goal to provide access to preventive oral care services^25^. Race has been reported to be one of the risk factors for periodontitis^2,5,12^. We found that compared to white participants, black, Hispanic, and participants of other races were associated with an increased odd of PD^2,5,7,12^. This finding aligned to a well-known periodontal study in the US^5^ that Hispanics had the highest prevalence of periodontitis (63.5%), followed non-Hispanics blacks (59.1%), non-Hispanic Asian Americans (50.0%). Whites had the lowest prevalence of PD (40.8%)^5^. Non-whites also had higher clinical loss attachment and periodontal probing depth^2^.

## Strengths and limitations

The main strengths of the present study stem from its reliance on the use of clinical data from dental examinations using standardized measurements provided by CDC/AAP and the definition of 3-category DM status that combined self-reported DM diagnosis and the laboratory report of glycemic control (HbA_1c_). This classification was derived to avoid redundancy in using both binary DM status (yes = 1, no = 0) and HbA_1c_ level (< 9% = 0, ≥ 9% = 1) variables simultaneously. Importantly, the 3-category DM status can measure the severity of the participant’s DM status more parsimoniously and precisely. An additional strength of the present study is that the sample analyzed (including adults aged ≥ 30 years) was representative of the US population, and the prevalence of PD and DM were consistent with previous reports. For example, Eke et al. used the 2009–2014 NHANES data to estimate that 42% of US adults aged 30 years and older had periodontitis^25^. The authors also reported that 9.6% of US adults had DM.

Limitations include the cross-sectional study design, which prevents us from examining causal relationships between PD, DM, and other risk factors using the NHANES data. Importantly, the NHANES questionnaire did not capture the participants’ treatment of PD, DM, and other known risk factors. A key limitation of NHANES data is that types of DM were not differentiated in NHANES, making it difficult to delineate between type 1 DM and type 2 DM^2^. Also, the 2009–2014 NHANES data were collected 10 years ago. However, it is the most recent U.S. national survey that included clinical examination data for dental conditions and laboratory report of HbA_1c_. People with incomplete oral examinations, and nursing home residents were excluded, which may result in potential selection bias.

## Future studies

Forthcoming studies should use longitudinal data to examine the causal relationship between glycemic control in DM and PD severity using the CDC/AAP case definitions of mild, moderate, and severe. A great need exists for longitudinal studies to analyze the effects of preventive dental care for people with systemic conditions (e.g., DM), especially older adults and racial minorities.

## Conclusions

The present study uses a sample of adults in the US and shows that uncontrolled DM was significantly associated with higher odds of PD compared to those with controlled and no DM in the US, particularly among those with low income and older age. Future investigations should explore the mechanisms through which uncontrolled DM increases the odds of PD. Ultimately, these relationships should be evaluated longitudinally.

## Data Availability

The NHANES data that support the findings of the present study are publicly available at the CDC website [https://wwwn.cdc.gov/nchs/nhanes/default.aspx], reference number^13,15^.
